# Subcellular Protein Localization by Using a Genetically Encoded Fluorescent Amino Acid

**DOI:** 10.1002/cbic.201100282

**Published:** 2011-06-16

**Authors:** Godefroid Charbon, Eric Brustad, Kevin A Scott, Jiangyun Wang, Anders Løbner-Olesen, Peter G Schultz, Christine Jacobs-Wagner, Eli Chapman

**Affiliations:** a Department of Molecular, Cellular, and Developmental Biology, KBT 1032, Yale UniversityNew Haven, CT 06520 (USA); b Department of Science, Systems and Models, Roskilde UniversityBuilding 18.1, 4000 Roskilde (Denmark); c Department of Chemistry, The Scripps Research InstituteSR202, 10550 North Torrey Pines Road, La Jolla, CA 92037 (USA); d Department of Molecular Biology, The Scripps Research InstituteMB46, 10550 North Torrey Pines Road, La Jolla, CA 92037 (USA); e Howard Hughes Medical InstituteNew Haven, CT 06510 (USA); f Microbial Pathogenesis Section, Yale School of MedicineNew Haven, CT 06510 (USA); g Present address: National Key Laboratory of Biomacromolecules, Institute of Biophysics, Chinese Academy of SciencesBeijing 100101 (China)

**Keywords:** fluorescent probes, protein modifications, proteins, synthetic biology, transfer RNA

The use of fluorescent protein fusions has revolutionized cell biology by allowing exploration of proteins in their native context. However, the utilization of current techniques is limited by the size and placement of the fused fluorescent protein. This is especially true for proteins that oligomerize or assemble into large complexes in which the fluorescent protein fusion can lead to improper assembly and/or function. To circumvent this problem, we report a novel technique for fluorescent labeling of proteins, in vivo. The unique potential of this technique lies in the ability to place a very small fluorescent tag virtually anywhere along a chosen protein sequence,^1^, ^2^ thereby minimizing the risk of affecting protein function. To illustrate the utility of this method, we have genetically encoded a single unnatural fluorescent amino acid in the sequence of the bacterial tubulin, FtsZ. This resulted in the production of a functional protein that could be visualized, in vivo.

The aim of this study was to explore novel methods of labeling proteins for in vivo localization studies that do not perturb protein function or structure. To this end, we have exploited a technique that allows unnatural amino acids with novel properties, in our case a fluorescent, coumarin-derived amino acid (CouAA; Figure 1 A), to be encoded into a given sequence.^1^, ^2^ The technique uses an orthogonal tRNA/aminoacyl-tRNA synthetase pair that transfers a defined unnatural amino acid to a growing polypeptide chain when nonsense amber codons (UAG)^1^ are present in the coding sequence. Specifically, we have used an archaebacteria amber suppressor tRNA (*Mj*-tRNA)/aminoacyl-tRNA synthetase (*Mj*-aaRS) pair (from *Methanococcus jannaschi*) that does not interact with endogenous *E. coli* aminoacyl-tRNA synthetases or tRNAs.^1^ This aaRS (CouRS) was then evolved by using a two-step selection process to specifically recognize CouAA and not an endogenous host amino acid. As a consequence, by simply inserting an amber stop codon in the desired gene sequence, an unnatural amino acid will be introduced in the corresponding translated protein (see cartoon in the Supporting Information). Conveniently, endogenous amber codons in *E. coli* and other organisms are likely poorly recognized by the *Mj*-tRNA, so that the expression of genes terminated by amber codons is apparently not affected. Although the exact mechanisms are not understood, it is known that the termination and suppression processes are influenced by sequences adjacent to the amber codon.^3^–^6^

**Figure 1:**
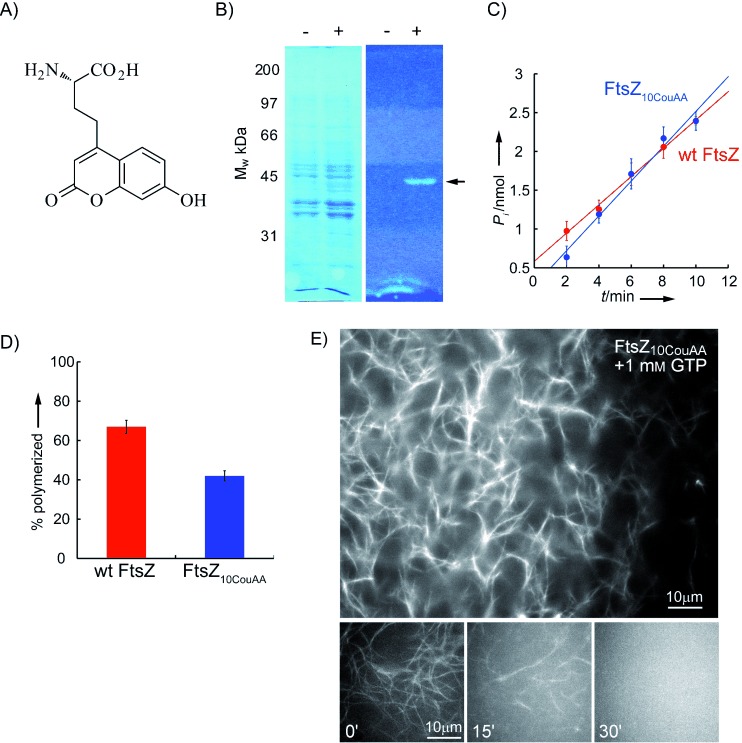
The biochemical properties of FtsZ_10CouAA_ are similar to that of wild type (wt). A) The unnatural amino acid, (*S*)-1-carboxy-3-(7-hydroxy-2-oxo-2*H*-chromen-4-yl)propan-1-aminium (CouAA). B) Protein extracts of cells grown with (+) or without (−) CouAA (1 mm) to visualize FtsZ_10CouAA_ expression, were separated by SDS-PAGE. FtsZ_10CouAA_ (arrowhead) was visualized with a 305 nm UV transilluminator (right-hand panel). The gel was then stained with Coomassie (left-hand panel). C) GTPase activity of purified His-tagged FtsZ and FtsZ_10CouAA_. D) Protein concentration after sedimentation assay by using purified His-tagged protein preparations of FtsZ and FtsZ_10CouAA_. GTP (2 mm) was used to initiate polymerization of FtsZ (5 μm) at pH 6.5 prior to centrifugation. E) Visualization of His-tagged FtsZ_10CouAA_ polymerized in the presence of GTP (1 mm). The lower panel shows a time course of FtsZ_10CouAA_ depolymerization, 0, 15, and 30 min after addition of GTP.

To demonstrate that this system can be effectively used as a means to visualize the in vivo subcellular location of a CouAA-labeled protein in bacteria, we chose to label the bacterial tubulin homologue FtsZ, which has been extensively studied both in vitro and in vivo. FtsZ assembles into a contractile ring (visible by fluorescence microscopy as a midcell band called Z-ring) during cytokinesis.^7^–^9^ But fusion of FtsZ to a fluorescent protein impairs its cellular function, a well-known problem for cytoskeletal proteins. Consequently, to date, all the reported FtsZ–fluorescent protein fusions have been shown to be nonfunctional on their own and must be produced in the presence of an untagged FtsZ copy for normal cell function.^10^ Thus, in this context, the FtsZ–fluorescent protein fusions merely label the endogenous, untagged FtsZ structure. While this approach has proven to be sufficient to generate considerable insight into FtsZ cellular function, it remains an imperfect artifice that might fail in the case of other oligomer-forming proteins or proteins forming macromolecular complexes. To visualize FtsZ in *E. coli*, we substituted the tenth amino acid (Asp10) for CouAA (FtsZ_10CouAA_). Asp10 is located in a disordered N-terminal segment with no known function and is likely to be surface exposed.^7^

To characterize the specificity of CouAA incorporation, His-tagged FtsZ_10CouAA_ was expressed in BL21(DE3) *E. coli* cells transformed with two plasmids: one constitutively expressed the evolved *Mj*-tRNA (pBKcouRS) and the other one (pBADJYftsZH6D10TAG) constitutively expressed the evolved tRNA synthetase (CouRS) and the mutated *ftsZ* gene under an arabinose inducible promoter. SDS-PAGE analysis (Figure 1 B) of total cell extract of cells grown in the absence or presence of CouAA (1 mm) in the growth medium to express FtsZ_10CouAA_, showed a unique band at approximately 40 kDa only with added CouAA. This band corresponds to the size of FtsZ and is fluorescent upon UV excitation and shows that the CouAA is specifically incorporated in FtsZ_10CouAA_.

The His-tagged FtsZ_10CouAA_ was then over-expressed and purified by using nickel metal affinity resin in order to characterize its in vitro properties. A standard malachite green assay was used to measure GTPase activity by using FtsZ (5 μm) and GTP (1 mm).^11^ The GTPase activity of His-tagged FtsZ_10CouAA_ was compared to wild-type His-tagged FtsZ and showed similar activity (Figure 1 C). Next, the ability of FtsZ to polymerize was measured by using a sedimentation assay: GTP (2 mm) was added to FtsZ (5 μm) at pH 6.5 and the resulting polymer was collected by centrifugation and quantified by using a Bradford assay.^12^ Figure 1 D shows that FtsZ_10CouAA_ is indeed able to polymerize, albeit to a slightly lesser extent than wild-type FtsZ. This difference might be due to a slightly higher rate of GTP hydrolysis exhibited by the FtsZ_10CouAA_ protein preparation leading to modest depolymerization during centrifugation after the GTP was depleted.^13^ Finally, the ability of FtsZ_10CouAA_ to polymerize was assessed directly by visualizing polymer formation by fluorescence microscopy. As shown in Figure 1 E, FtsZ_10CouAA_ formed an extended meshwork of polymers in the presence of GTP. As expected, depletion of GTP in the buffer resulted in depolymerization of the FtsZ_10CouAA_ structures over time. Thus, by and large, FtsZ_10CouAA_ behaves like its wild-type counterpart, in vitro.

To visualize FtsZ_10CouAA_ in growing *E. coli*, we used MC1000 cells harboring two expression plasmids, pBKcouRS and pBADJYftsZD10TAG (expressing the mutated *ftsZ* gene without His-tag). FtsZ_10CouAA_ subcellular localization was imaged after addition of arabinose to growth media supplemented with CouAA (1 mm, Figure 2 A); cells were washed twice with M9 minimal media (lacking CouAA) prior to visualization in order to remove unincorporated CouAA. After 40 min of induction clear Z-rings could be visualized. After 120 min FtsZ_10CouAA_ was clearly over-expressed as it formed helical structures rather than Z-rings and cell division was perturbed. Both of these phenotypes are known to result from FtsZ over-expression.^14^–^16^ Furthermore, when cell division was blocked by using the β-lactam, cephalexin, FtsZ_10CouAA_ formed multiple independent Z-rings as expected (Figure 2 B). GFP–FtsZ fusions have been used in the past to demonstrate the dynamic nature of the Z-ring. Similarly, we assayed the dynamics of FtsZ_10CouAA_ Z-rings by fluorescence recovery after photobleaching (FRAP) microscopy. Cells were first treated with cephalexin prior to FRAP analysis in order to block cell division for greater spatial resolution. We obtained an average half-time recovery of 12(±5) s (Figure 3), which is in agreement with results obtained by others using GFP–FtsZ variants.^17^ These results demonstrate that the incorporation of a single fluorescent amino acid into a protein sequence is sufficient to observe protein subcellular localization, in vivo.

**Figure 2:**
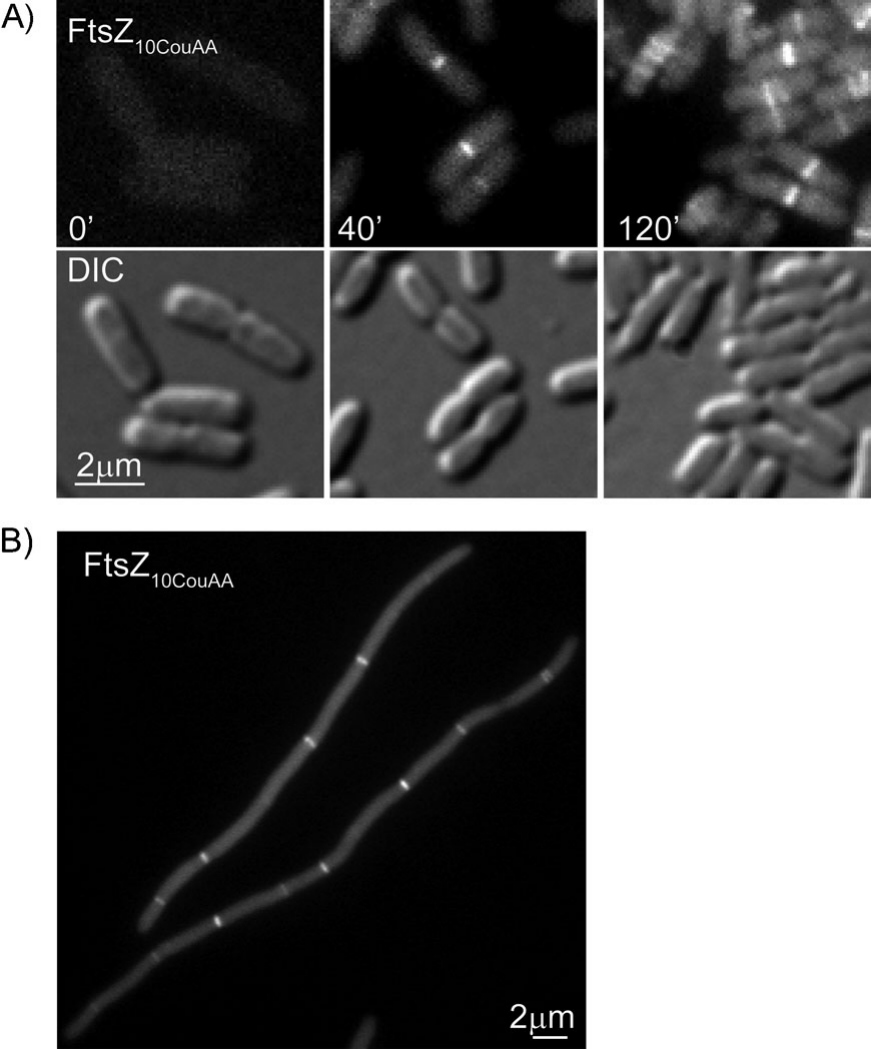
Visualization of FtsZ_10CouAA_ subcellular localization. A) FtsZ_10CouAA_ subcellular localization. Fluorescence (top panels) and DIC (lower panels) images after 0, 40, and 120 min postinduction of FtsZ_10CouAA_ synthesis are shown. B) Fluorescence image of FtsZ_10CouAA_ in *E. coli* cells treated with cephalexin.

**Figure 3:**
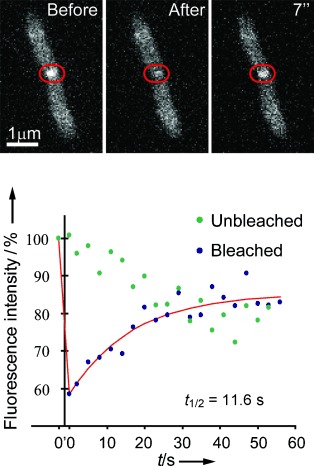
The in vivo dynamic properties of FtsZ_10CouAA_. Fluorescence recovery after photobleaching (FRAP). FtsZ_10CouAA_ Z-ring prior to, immediately after, and 7 s postphotobleaching. The graph represents the data corrected for photobleaching due to image acquisition for unbleached (green) and bleached (blue) regions; the red line represents the theoretical recovery curve fit. FtsZ_10CouAA_ half-time recovery is 12(±5) s (mean ±standard deviation); 11.6 s in the example shown.

As mentioned above, one major problem with existing methods for in vivo localization studies of FtsZ–fluorescent protein constructs is that these constructs are nonfunctional and cannot substitute for the wild-type FtsZ protein. This is due to the presence of the sterically demanding terminal fluorescent protein fusion. FtsZ_10CouAA_ does not harbor tags or fusions, only a single fluorescent amino acid. In addition, purified FtsZ_10CouAA_ showed biochemical properties similar to the wild type, in vitro. This led us to believe that FtsZ_10CouAA_ might be functional, in vivo. To test this, we generated *E. coli* cells producing FtsZ_10CouAA_ as the only source of FtsZ. A deletion of the chromosomal *ftsZ* gene^18^ was introduced into MC1000 cells harboring the required orthogonal *Mj*-tRNA/CouRS pair (pBADJYftsZD10TAG and pBKcouRS). The recipient strain was grown in media supplemented with L-arabinose (0.2 %) and CouAA (1 mm).

The cells appeared approximately like the wild type (Figure 4 A), with Z-rings clearly visible (insert). The size of the cells varied slightly more than the wild type, most probably because of inconsistent expression of FtsZ_10CouAA_ from the arabinose promoter—too low or too high FtsZ expression directly influences cell size.^14^ In stark contrast, when the cells were shifted to a medium that did not contain the arabinose inducer (Figure 4 B and D), the cells became filamentous as is expected in the absence of FtsZ. Likewise, cells grown in the absence of CouAA, but with arabinose, became filamentous albeit more slowly than when FtsZ_10CouAA_ expression was turned off (Figure 4 C; see also movies S1 and S2 in the Supporting Information). This slight difference most probably reflects more rapid cellular elimination of arabinose than CouAA or perhaps the presence of a weak, endogenous, amber suppressor, although no such product was detected by SDS-PAGE or mass spectroscopy. Nevertheless, our results show that FtsZ_10CouAA_ can substitute for wild-type FtsZ, in vivo.

**Figure 4:**
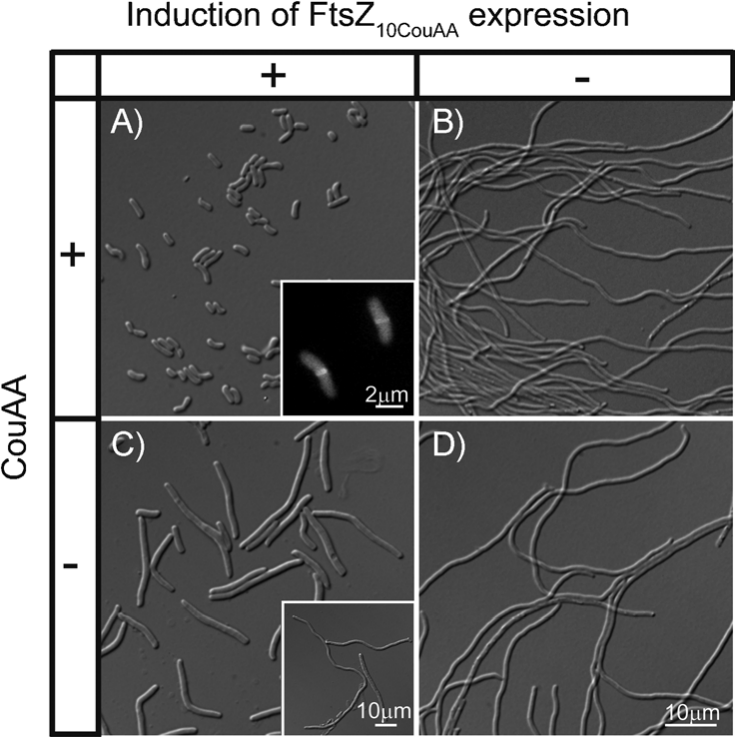
FtsZ_10CouAA_ is functional, in vivo. A) *E. coli* FtsZ^−^ cells expressing FtsZ_10CouAA_ as the sole source of FtsZ were grown in the presence of CouAA (1 mm) and L-arabinose (0.2 %) to maintain FtsZ_10CouAA_ expression; insert: fluorescence image of cells grown with L-arabinose and CouAA. B)–D) The culture was split in four and incubation was continued for 5 h in the presence and absence of CouAA and of L-arabinose, as indicated in the panels. Insert: C) Cells incubated for 12 h in the absence of CouAA.

Many proteins form large assemblies, either homo- or hetero-oligomeric, that prohibit sterically demanding appendages. Here we show that genetically encoding an unnatural amino acid provides an alternative methodology when conventional techniques fail. We successfully visualized FtsZ by genetically encoding a single fluorescent amino acid into its sequence. We also showed that the fluorescent CouAA was incorporated specifically into the protein sequence, and that the resulting FtsZ variant retained its functionality. While some restrictions, such as low efficiency of CouAA incorporation and the photophysical properties of the fluorophore, might apply,^19^ it is manifest that the system is sensitive enough to specifically visualize the subcellular localization and dynamics of a protein complex. The advantages of this technique are the small size of the fluorescent label (one amino acid) and the fact that the CouAA label in theory can be positioned anywhere in the protein of interest (although context sequences will influence the efficiency of amino acid incorporation^3^, ^4^, ^20^). Thus, this technique offers unique opportunities to harvest previously unobtainable data. Although the present unnatural amino acid incorporation system is designed for bacteria, new systems have been developed for other organisms.^21^

## Experimental Section

**FtsZ_10CouAA_** **visualization:** Cells were visualized by using a DAPI filter set (excitation: 345 nm; emission: 458 nm) with a Nikon E1000 microscope equipped with a 100× DIC objective and a Hamamatsu Orca-ER LCD camera or a Nikon E80i microscope with a DIC 100× objective and an Andor iXon+ camera. Photobleaching experiments were performed by using a Photonic instruments Micropoint laser system equipped with a 364 nm laser dye, set at 10 to 15 pulses with maximum laser intensity. Images were taken and processed with Metamorph (6.1r0 or 7.1.4) and ImageJ software.

Purified His-tagged FtsZ_10CouAA_ was polymerized in MES (50 mm, pH 6.5), KCl (100 mm), MgCl_2_ (10 mm) supplemented with GTP (1 mm) prior to immobilization on polylysine-treated cover-slips. FtsZ_10CouAA_ polymers were visualized by using a Leica DM5000B microscope equipped with a 100× phase contrast objective and a Leica DFC 350 FX camera. Images were taken and processed with Micro-Manager 1.4 software.

**FtsZ GTPase activity assays:** GTPase activity was measured in HEPES (50 mm, pH 7.2), MgCl_2_ (10 mm), KCl (200 mm), with 5 μm wild-type His-tagged FtsZ or His-tagged FtsZ_10CouAA_, and initiated by addition of GTP (1 mm). Activity was assayed by using a standard malachite green assay.^22^

**FtsZ sedimentation assay:** Polymerization of FtsZ was carried out as reported previously.^12^ Wild-type His-tagged FtsZ and His-tagged FtsZ_10CouAA_ (5.0 μm) were polymerized in MES (50 mm, pH 6.5), KCl (200 mm), MgCl_2_ (10 mm) containing GTP (5 mm) at 37 °C. The polymers were collected by centrifugation at 227 000 *g* for 30 min at 30 °C. The protein concentration in the supernatant was measured by a Bradford assay by using BSA as a standard to calculate the percent polymerized.

## Notes

Detailed facts of importance to specialist readers are published as ”Supporting Information”. Such documents are peer-reviewed, but not copy-edited or typeset. They are made available as submitted by the authors.
