# Supplementary material for: Subcellular Protein Localization by Using a Genetically Encoded Fluorescent Amino Acid
Source: Chembiochem. 2011 Jun 16;12(12):1818–21. doi: 10.1002/cbic.201100282 (PMC3175735; doi:10.1002/cbic.201100282)
Supplement: Supplementary file 1 [file cbic0012-1818-SD1.pdf]

CHEMBIOCHEM

Supporting Information

© Copyright Wiley-VCH Verlag GmbH & Co. KGaA, 69451 Weinheim, 2011

Subcellular Protein Localization by Using a Genetically Encoded Fluorescent Amino Acid

Godefroid Charbon,[a, b] Eric Brustad,[c] Kevin A. Scott,[d] Jiangyun Wang,[c, g] Anders Løbner-Olesen,[b] Peter G. Schultz,[c] Christine Jacobs-Wagner,[a, e, f] and Eli Chapman*[d]

cbic_201100282_sm_miscellaneous_information.pdf
cbic_201100282_sm_mov1.mov
cbic_201100282_sm_mov2.mov

Supplemental Cartoon

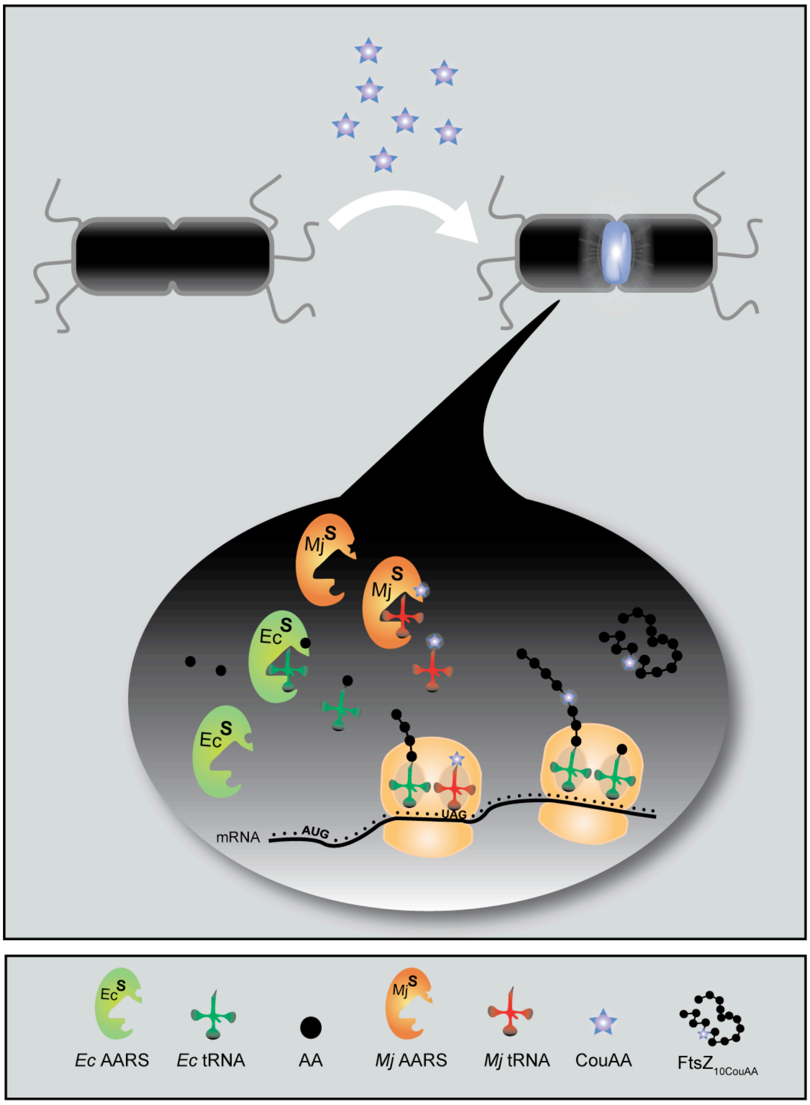

Supplemental Methods

Bacterial strains.

MC1000 (*araD139 Δ(ara-leu)* 7679, *gal U, galK, Δ(lac)X74,* Str ^{r}, *thi*) and DH10B (F$^-$ mcrA Δ(*mrr-hsd*RMS-*mcr*BC) ϕ80d*lacZ*ΔM15 Δ*lac*X74 *end*A1 *rec*A1 *deo*R Δ(*ara,leu*)7697 *ara*D139 *gal*U *gal*K *nup*G *rps*L λ^-) cells were used for $FtsZ_{10CouAA}$ visualization and $FtsZ_{10CouAA}$ overproduction, respectively. We observed strain variation in $FtsZ_{10CouAA}$ expression, possibly reflecting the strength of the arabinose promoter in the strains (DH10B=BL21> MC1000 >MG1655).

The strain expressing $FtsZ_{10CouAA}$ as the sole source of FtsZ was constructed as follows. A P1 lysate from strain JKD7-1(pKD4)[1] was used to transduce a deletion of *ftsZ* into a MC1000 strain harboring the required suppression plasmids (pBADJYftsZD10TAG and pBKcouRS).

Plasmids.

pBKcouRS was selected from a library of *M. jannaschi* tyrosyl tRNA synthetase (TyrRS) mutants as reported previously.[2] pBADJYftsZD10TAG was constructed as follows: the *ftsZ* gene was amplified from *E. coli* genomic DNA using primers ftsZH6_F (5'-ggcattcatatgcaccatcaccatcaccattttgaaccaatggaacttaccaat-3') and ftsZ_R (5'-gcagccggatccttaatcagcttgcttacgc-3'). The PCR product was subsequently digested with NdeI and BamHI and cloned into pET11a digested with the same enzymes to create pET11-H6ftsz. *ftsZ* was amplified from pET11-H6ftsZ using primers ftsZ_NcoI_F (5'-ggagataccatggagtttgaaccaatggaacttaccaattag-3') and ftsZ_NdeI_R (5'-gcagcccatatgttaatcagcttgcttacgc-3'). The PCR product was digested with NcoI and NdeI and cloned into pBADJY[2] digested with the same enzymes to create pBADJYftsZD10TAG.

pBADJYH6ftsZD10TAG was created similarly by amplification of *ftsZ* from pET11-H6ftsZ using primers H6_ftsZ_F (5'-ggagataccatgggccaccatcaccatcaccat-3') and ftsZ_NdeI_R (5'-gcagcccatatgttaatcagcttgcttacgc-3') . The PCR product was digested with NcoI and NdeI and cloned into pBADJY digested with the same enzymes.

FtsZ mutagenesis was carried out using the modified quick change procedure of Reymond and coworkers[3] using primers D10TAG_F (5'-ccaattag gcggtgattaaagtcatcggcgtcg-3') and D10TAG_R (5'-accgcctaattggtaagttccattggttcaaac-3').

Growth conditions.

Luria–Bertani (LB) medium or M9 minimal medium supplemented with 10µg/ml thiamine, 0.2% glycerol and 0.1% casamino acids was used for visualization of $FtsZ_{10CouAA}$. LB Medium was supplemented with 50µg/ml kanamycin, 10µg/ml tetracycline and 100µg/ml ampicillin when appropriate. M9 medium was supplemented with 25µg/ml kanamycin and 5µg/ml tetracycline, when appropriate. $FtsZ_{10CouAA}$ expression was induced by addition of 0.2% L-arabinose and 1 mM CouAA when cell cultures reached an $O.D._{600} = 1.0$ or $O.D._{450} = 0.2$ in LB and M9, respectively. The culture tubes were shielded from light with aluminium foil to avoid photo-degradation of the CouAA. In LB, we could only observe induction of $FtsZ_{10CouAA}$ synthesis in late log phase growing culture whereas induction could be readily achieved in M9 minimal medium during log phase.

For His-tagged $FtsZ_{10CouAA}$ overproduction, cells were grown in Terrific Broth (TB).

$FtsZ_{10CouAA}$ visualization.

Cells were grown in presence of 1mM CouAA, 0.2% L-arabinose, for a duration of 40 to 120 minutes in LB and for a duration of 120 minutes in M9. Cephalexin (10µg/ml) was added for a duration of 2 hours when relevant. Cells were then pelleted and resuspended twice in M9 medium in order to remove the non-incorporated CouAA and auto-fluorescent compounds present in the growth media. Cells were then deposited on M9-based agarose (1%) pad for visualization.

Cells were visualized using a DAPI filter set, excitation 345 nm and emission 458 nm, using a Nikon E1000 microscope equipped with a 100X DIC objective and a Hamamatsu Orca-ER LCD camera or using a Nikon E80i microscope with a DIC 100X objective and an Andor iXon+ camera. Photobleaching experiments were performed using a Photonic Instruments Micropoint Laser system equipped with a 364nm Laser Dye, set at 10 to 15 pulses with maximum laser intensity. Images were taken and processed with Metamorph (6.1.0 or 7.1.4) and ImageJ software.

Purified His-tagged $FtsZ_{10CouAA}$ was polymerized in 50mM MES pH 6.5, 100mM KCl, 10mM $MgCl_2$ supplemented with 1mM GTP prior to immobilization on polylysine-treated cover slips. $FtsZ_{10CouAA}$ polymers were visualized using a Leica DM5000B microscope equipped with a 100x phase contrast objective and a Leica DFC 350 FX camera. Images were taken and processed with Micro-Manager 1.4 software.

His-tagged FtsZ protein purification.

Wt FtsZ harboring an N-terminal hexahistidine tag was produced in BL21(DE3) cells and purified by NiNTA agarose chromatography. Cells overproducing FtsZ were resuspended in buffer A (50 mM Hepes pH 7.2, 0.1 mM EDTA, 500 mM NaCl, and 0.05% Triton X-100), lysed with a microfluidizer (Microfluidics Corp.), and clarified by centrifugation. The resulting supernatant was

loaded on a charged NiNTA HiTrap column (GE Bioscience) equilibrated in Buffer A, washed with 10 column volumes (CV) buffer A, and eluted with a gradient of 0-200 mM imidazole in buffer A over 20 (CV). Fractions with FtsZ were pooled and dialyzed against 50 mM HEPES pH 7.2, 0.1 mM EDTA, and 10% glycerol. The dialyzed sample was concentrated, flash frozen and stored at -80°C until needed. FtsZ10CouAA was produced in and purified from DH10B cells harbouring pBKcouRS and pBADJYH6FtsZD10TAG following a similar protocol, except light was excluded during growth and purification. DH10B cells were grown to OD_{600}=1.1 in 250 mL of TB at which time L-arabinose and CouAA were added to 0.2% and 1 mM final concentrations, respectively. The culture was then grown for 3 hours and His-tagged $FtsZ_{10CouAA}$ was purified as for wt FtsZ.

FtsZ GTPase activity assays.

GTPase activity was measured in 50 mM HEPES pH 7.2, 10 mM $MgCl_2$, 200 mM KCl, with 5 μM wt His-tagged FtsZ or His-tagged $FtsZ_{10CouAA}$, and initiated by addition of 1 mM GTP. Activity was assayed using a standard malachite green assay.[4]

FtsZ sedimentation assay.

Polymerization of FtsZ was carried out as reported previously.[5] Wt His-tagged FtsZ and His-tagged $FtsZ_{10CouAA}$ (5.0 μm) were polymerized in 50 mM MES pH 6.5, 200 mM KCl, 10 mM $MgCl_2$ containing 5 mM GTP at 37 °C. The polymers were collected by centrifugation at 227,000 × g for 30 minutes at 30 °C. The protein concentration in the supernatant was measured by a Bradford assay using BSA as a standard to calculate the percent polymerized.

CouAA Synthesis

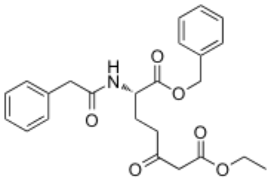

Synthesis of (S)-1-benzyl 7-ethyl 5-oxo-2-(2-phenylacetamido)heptanedioate. Mono-ethyl malonate (1.75 mL, **14**.8 mmol) was dissolved in 25 mL anhydrous tetrahydrofuran (THF) at 23°C. Solid Mg(OEt)2 (850 mg, 7.4 mmol) was added and the reaction stirred at 23°C for 60 minutes. Z-Glu-OBzl (5 g, 13.5 mmol) was dissolved in 50 mL anhydrous THF at 23°C. Carbonyldiimidazole (2.4 g, 14.8 mmol) was added and the reaction stirred at 23°C for 60 minutes. The mono-ethyl magnesium salt was transferred to the activated Z-Glu-OBzl and the reaction stirred 8 hours at 23°C. The product was extracted with ether, washed with saturated NaHCO3, water, and brine. The residue was purified by flash chromatography on silica gel (ethyl acetate:hexanes 50:50) and concentrated on a rotary evaporator to afford a white solid in 93% yield. 1H-NMR (400 MHz, CDCl3) : δ 1.26 (t, J = 7.2, 3H), 1.9-2.01 (m, 1H), 2.1-2.28 (m, 1H), 2.5-2.7 (m, 2H), 3.37 (s, 2H), 4.18 (q, J = 7.2, 2H), 4.41 (s, 1H), 5.11 (s, 2H), 5.18 (s, 2H), 5.44 (d, J = 8, 1H), 7.25-7.41 (m, 10H). LC-MS (ESI) calcd for $C_{24}H_2NO_7$ (M^+ H^+) 442.18, obsd. 442.0.

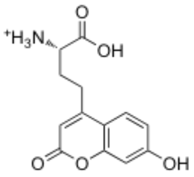

Synthesis of (S)-1-carboxy-3-(7-hydroxy-2-oxo-2H-chromen-4-yl)propan-1-aminium (CouAA). The compound was synthesized as reported previously.[6] 1H-NMR (DMSO): δ 2.06 (s, 2H), 2.70-2.90 (m, 2H), 3.82-4.0 (m, 1H), 6.06 (s, 1H), 6.75 (dd, J = 8.8, 2.4, 1H), 6.82 (d, J = 2.4, 1H), 7.62 (d, J = 8.8, 1H), 8.17 (s, 3H). LC-MS (ESI) calc.for $C_{13}H_{14}NO_5$ (M^+ H^+) 264.08 Da, obsd. 264.0 Da.

Movie legends

MovieS1: Time-lapse microscopy showing *E. coli* FtsZ^{-} cells expressing FtsZ$_{10CouAA}$ as the sole source of FtsZ

MC1000 Δ*ftsZ* harbouring the pBADJYftsZD10TAG and pBKcouRS plasmids grown in LB in the presence of 10mM CouAA, 0.2% L-arabinose were imaged on an LB agarose pad containing 10mM CouAA and 0.2% L-arabinose to maintain FtsZ$_{10CouAA}$ expression.

MovieS2: Time-lapse microscopy showing *E. coli* FtsZ^{-} cells upon cessation of FtsZ$_{10CouAA}$ expression.

MC1000 Δ*ftsZ* harbouring the pBADJYftsZD10TAG and pBKcouRS plasmids were first grown in LB in the presence of 1mM CouAA and 0.2% L-arabinose to maintain FtsZ$_{10CouAA}$ expression. Cells were washed and deposited on an LB agarose pad containing 0.2% L-arabinose but no CouAA, to arrest FtsZ$_{10CouAA}$ expression.

Reference List

[1] K. Dai, J. Lutkenhaus *J. Bacteriol.* **1991,** ***173,*** 3500-3506.

[2] J. Wang, J. Xie, P. G. Schultz, *J. Am. Chem. Soc.* **2006,** *128*, 8738-8739.

[3] L. Zheng, U. Baumann, J. L. Reymond, *Nucleic Acids Res* **2004,** *32,* e115.

[4] T. P. Geladopoulos, T. G. Sotiroudis, A. E. Evangelopoulos, *Anal. Biochem.* **1991,** *192,* 112-116.

[5] A. Mukherjee, J. Lutkenhaus, *Methods Enzymol.* **1998,** *298*, 296-305.

[6] M. P. Brun, L. Bischoff, C. Garbay, *Angew. Chem. Int. Ed. Engl.* **2004,** *43*, 3432-3436.
